# Cardiopulmonary Exercise Testing in Dysfunctional Breathing: A Case Series of Five Young Women

**DOI:** 10.1002/rcr2.70336

**Published:** 2025-08-31

**Authors:** Arup Haldar, Raja Dhar, Diptendu Dey, Amrita Bhattacharya, Beauty Biswas, Shyam Krishnan

**Affiliations:** ^1^ The Calcutta Medical Research Institute Hospital Kolkata India; ^2^ Department of Pulmonology The Calcutta Medical Research Institute Hospital Kolkata India; ^3^ Respiratory Medicine The Calcutta Medical Research Institute Hospital Kolkata India; ^4^ Respiratory Intensive Care Unit The Calcutta Medical Research Institute Hospital Kolkata India

**Keywords:** cardiopulmonary exercise testing, dysfunctional breathing, dyspnoea, plethysmography, young adults

## Abstract

Dysfunctional breathing (DB) is an underdiagnosed condition in young patients presenting with unexplained exertional breathlessness. Cardiopulmonary exercise testing (CPET) provides valuable diagnostic insights in these challenging cases. We present a case series of five young women (mean age 23.2 years) who underwent comprehensive evaluation including CPET and body plethysmography for persistent exertional dyspnea. All patients demonstrated characteristic CPET findings of oscillatory breathing patterns, preserved ventilatory reserve, elevated VE/VCO_2_ slope, and absence of oxygen desaturation. Body plethysmography revealed consistently elevated residual volumes (162%–296% predicted) and RV/TLC ratios (40.39%–50.21%). CPET is crucial for diagnosing DB in young patients with unexplained dyspnea. The consistent plethysmography abnormalities suggest potential underlying airway pathology or maladaptive breathing patterns.

## Graphical abstract

This case series highlights CPET's critical role in diagnosing dysfunctional breathing in young women with unexplained exertional dyspnea, identifying characteristic oscillatory breathing patterns, preserved ventilatory reserve, and ventilatory inefficiency as objective evidence.

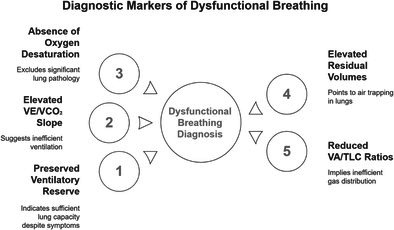

## 1 Introduction

Dysfunctional breathing (DB) represents a spectrum of breathing pattern disorders that can significantly impact quality of life, particularly in young women. The condition is characterised by altered breathing patterns that are inappropriate for metabolic demands, often leading to symptoms of dyspnea, chest discomfort, and systemic manifestations [1]. Despite its clinical significance, DB remains underdiagnosed, partly due to the lack of standardised diagnostic criteria and the overlap of symptoms with other respiratory and cardiac conditions [2].

Cardiopulmonary exercise testing (CPET) has emerged as a valuable tool in the evaluation of unexplained dyspnea, providing objective assessment of the integrated response of the cardiovascular, respiratory, and metabolic systems during exercise. In patients with DB, CPET can reveal characteristic patterns that help differentiate this condition from organic cardiopulmonary disease [3].

This case series presents five young women with persistent exertional dyspnea in whom CPET played a crucial role in establishing the diagnosis of DB (Table 1).

**TABLE 1:** Patient demographics and plethysmography parameters.

	Age	Sex	BMI	FEV1	FVC	Ratio	DLCO	Va/TLC	TLC	RV	RV/TLC
Patient 1	22	F	27.2	79%	76%	92.6	80%	0.74	114%	211%	50.21
Patient 2	16	F	25.9	86%	86%	90	71%	0.7	111%	182%	40.39
Patient 3	17	F	22.1	89%	91%	89.4	91%	0.7	150%	296%	50.11
Patient 4	28	F	26.3	75%	73%	85.3	86%	0.71	108%	181%	46.83
Patient 5	33	F	38.5	81%	79%	83.4	72%	0.7	102%	162%	46.89

## 2 Case Series

We conducted a retrospective case series of five young women (mean age 23.2 years) presenting with unexplained exertional dyspnea. All participants underwent a standard cardiopulmonary exclusion workup (clinical examination, ECG, echocardiography, chest x‐ray, spirometry). Inclusion required findings to be unremarkable, with no evidence explaining symptoms. Subsequently, comprehensive CPET and body plethysmography were performed to characterise ventilatory patterns and lung volumes. Data were analysed descriptively.

### 2.1 Case 1: 22‐Year‐Old Female With Post‐Viral Dyspnea

A 22‐year‐old female (BMI 27.2 kg/m^2^) presented with an 8‐week history of exertional dyspnea (mMRC grade 2), chest tightness, and palpitations following a viral upper respiratory tract infection. She reported significant functional limitation, being unable to climb stairs without severe breathlessness.

Spirometry showed forced expiratory volume in 1 s (FEV_1_) 79% predicted, forced vital capacity (FVC) 76% predicted, with FEV_1_/FVC ratio of 92.6%.

Exercise testing revealed oscillatory breathing patterns throughout exercise, preserved ventilatory reserve (51%), elevated minute ventilation/carbon dioxide production (VE/VCO_2_) slope (74), and no oxygen desaturation. Peak oxygen consumption was reduced relative to predicted values (39%).

Body plethysmography demonstrated elevated total lung capacity (114% predicted), markedly increased residual volume (RV) (211% predicted), and elevated RV/Total Lung Capacity (TLC) ratio (50.21%). Diffusing capacity of the lungs for carbon monoxide (DLCO) was at 80% predicted with alveolar volume (VA)/TLC ratio of 0.74.

Patient was diagnosed with DB and referred for breathing retraining therapy.

### 2.2 Case 2: 16‐Year‐Old Female With Performance Anxiety

A 16‐year‐old female athlete (BMI 25.9 kg/m^2^) developed progressive exercise intolerance (mMRC grade 2) over 6 weeks, with associated mood changes and generalised weakness. Previously active in competitive sports, she became unable to participate due to dyspnoea.

Spirometry showed FEV_1_ 86% predicted, FVC 86% predicted, with FEV_1_/FVC ratio of 90%.

Characteristic oscillatory breathing pattern was observed during exercise, with preserved ventilatory reserve (55%) and elevated VE/VCO_2_ slope (63.29). No evidence of cardiac limitation or oxygen desaturation.

Total lung capacity was 111% predicted with a residual volume of 182% predicted. RV/TLC ratio was elevated at 40.39%. DLCO was 71% predicted with a VA/TLC ratio of 0.70.

Diagnosed with DB secondary to performance anxiety and a breathing pattern disorder.

### 2.3 Case 3: 17‐Year‐Old Female With Severe Functional Limitation With Unclassified Airway Disease

A 17‐year‐old female (BMI 22 kg/m^2^) with a previous history of Allergic Rhinitis presented with severe exertional dyspnea (mMRC grade 3) of 12‐week duration, associated with chest pain and fatigue.

Spirometry showed FEV_1_ 89% predicted, FVC 91% predicted and FEV1/FVC ratio of 89.4.

Exercise testing demonstrated the oscillatory breathing pattern, with marked ventilatory inefficiency (VE/VCO_2_ slope 50.91) and preserved ventilatory reserve (44%). No cardiac limitation was identified.

Most abnormal lung volumes in the series with TLC 150% predicted and markedly elevated RV of 296% predicted. RV/TLC ratio was 50.11%. DLCO was normal at 91% predicted.

Severe DB with possible underlying airway dysfunction requiring comprehensive breathing rehabilitation.

### 2.4 Case 4: 28‐Year‐Old Female With Chronic Symptoms

A 28‐year‐old female (BMI 26.3 kg/m^2^) reported protracted exertional dyspnoea (mMRC grade 2) with recent worsening over 10 weeks. Symptoms included chest discomfort, palpitations, and exercise intolerance affecting daily activities.

Spirometry revealed FEV_1_ 75% predicted, FVC 73% predicted, with FEV_1_/FVC ratio of 85.3%.

Oscillatory breathing pattern with ventilatory inefficiency (VE/VCO_2_ slope 53.94) and preserved ventilatory reserve (42%). Exercise capacity was limited by subjective dyspnea rather than cardiac factors.

TLC 108% predicted with RV of 181% predicted. RV/TLC ratio was 46.83%. DLCO was at 86% predicted.

DB managed with breathing exercises and lifestyle modifications.

### 2.5 Case 5: 33‐Year‐Old Female With Metabolic Considerations

A 33‐year‐old female (BMI 38.5 kg/m^2^) developed progressive dyspnea (mMRC grade 2) worsened over 8 weeks, with associated gendered weakness and mood instability. Her higher BMI raised considerations of obesity‐related breathing disorders.

Sleep study was normal. Spirometry showed FEV_1_ 81% predicted, FVC 79%, and FEV_1_/FVC ratio of 83.4%.

Characteristic oscillatory breathing pattern was observed with ventilatory inefficiency (VE/VCO_2_ slope 43.06) with normal breathing reserve (43%). No evidence of obesity hypoventilation or cardiac limitation.

TLC 102% predicted with RV 162% predicted (lowest in the series but still significantly elevated). RV/TLC ratio was 46.89%. DLCO was 72% predicted.

DB with metabolic considerations, managed with a comprehensive approach including weight management and breathing retraining.

## 3 Discussion

**FIGURE 1:**
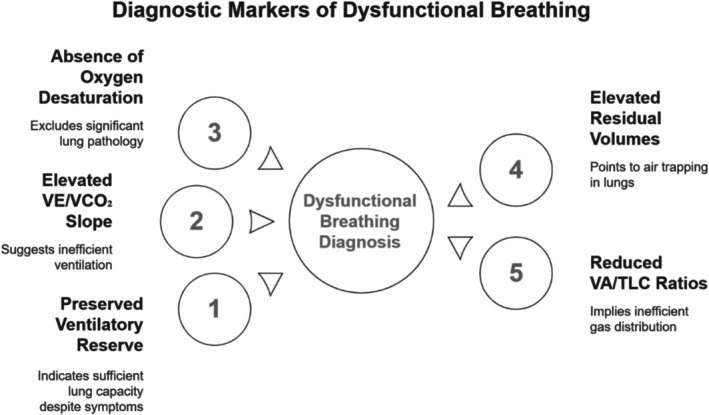
Diagnostic markers of dysfunctional breathing.

**FIGURE 2:**
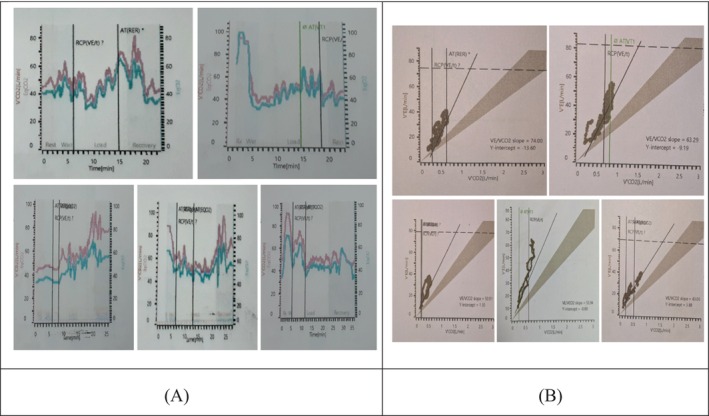
(A) Oscillatory breathing pattern in dysfunctional breathing. (B) VE/VCO_2_ slope in dysfunctional breathing.

This case series underscores the diagnostic utility of CPET in identifying DB, particularly in young women presenting with unexplained dyspnea. The consistent CPET findings across all five patients provide a robust profile highly suggestive of DB, differentiating it from cardiopulmonary organic disease (Figure 1). The most pathognomonic feature observed was the oscillatory or irregular breathing pattern during exercise, indicative of disrupted neuro‐respiratory control, often stress or hyperventilation‐driven (Figure 2A) [4]. Crucially, this occurred alongside a preserved ventilatory reserve (> 30%), demonstrating that ventilatory capacity was not the limiting factor despite significant dyspnea; instead, symptoms arose from maladaptive respiratory muscle recruitment and heightened perception of effort. Further supporting the diagnosis was the elevated VE/VCO_2_ slope (> 34), signifying ventilatory inefficiency—a core characteristic of DB (Figure 2B) [5]. The consistent absence of exercise‐induced oxygen desaturation effectively excluded significant intrinsic pulmonary pathology, reinforcing DB as a disorder primarily of ventilatory control without gas exchange impairment. As highlighted by Ionescu et al. [4], the co‐occurrence of these CPET markers (oscillatory pattern, elevated VE/VCO_2_, preserved ventilatory reserve) offers high specificity (89%) for DB.

An equally striking and consistent finding was the plethysmographic evidence of abnormal lung volumes. All patients exhibited markedly elevated residual volumes (RV) (162%–296% predicted) and RV/TLC ratios (40.39%–50.21%), alongside reduced VA/TLC ratios (0.70–0.74). While elevated RV and RV/TLC classically signify air trapping in obstructive diseases like asthma or COPD, the presence of normal spirometry in these DB patients points towards a different aetiology: maladaptive breathing mechanics. This pattern strongly suggests thoraco‐dominant breathing, where over‐reliance on accessory muscles compromises diaphragmatic efficiency and impairs complete exhalation. Frequent sighing or breath‐holding, common in DB, further disrupts expiratory flow, promoting functional residual capacity increase and chronic air trapping [6]. The reduced VA/TLC ratio implies inefficient gas distribution or micro‐atelectasis, likely stemming from chronic shallow breathing patterns reducing alveolar recruitment—a mechanism distinct from the restrictive defects seen in intrinsic lung diseases like Interstitial fibrosis. These plethysmographic abnormalities, reported in 30%–40% of DB patients (RV > 150% predicted), are frequently misinterpreted as occult obstruction but, in the context of normal spirometry and characteristic CPET findings, strongly support DB mechanics as the underlying cause [6]. They suggest a state of chronic air trapping resulting from inefficient breathing patterns, potentially compounded by subtle, undiagnosed airway hyperresponsiveness, though not meeting spirometric criteria for obstruction. Despite Ruane et al.'s finding of DB prevalence at 47% in severe asthma [7], the present study, conducted without bronchoprovocation testing, could not conclusively rule out underlying asthma or characterise the varied DB phenotypes outlined by these authors.

The consistent plethysmography abnormalities in our series raise important questions about the pathophysiology of DB. The elevated residual volumes may represent:

1. Subclinical airway disease: Airway dysfunction that is not detected by standard spirometry but becomes apparent on plethysmography.
2. Breathing pattern dysfunction: Chronic maladaptive breathing patterns leading to persistent air trapping and altered lung mechanics.
3. Inefficient gas distribution: A reduced VA/TLC ratio suggests inefficient gas distribution (e.g., micro‐atelectasis) from chronic shallow breathing impairing alveolar recruitment—distinct from true restrictive disease.

This case series highlights CPET's critical role in diagnosing DB in young women with unexplained exertional dyspnea, identifying characteristic oscillatory breathing patterns, preserved ventilatory reserve, and ventilatory inefficiency as objective evidence. A prospective multicentre study is warranted to validate these findings and confirm the generalisability of functional ventilatory impairment as a distinct phenomenon in DB.

## Author Contributions

All authors contributed to designing, executing, and writing the manuscript.

## Ethics Statement

The study was cleared by the Institutional Ethics Committee (ECR/132/Inst/WB/2013/RR‐19) at the Calcutta Medical Research Institute. Ref No: IEC/01/2025/APRV/03.

## Consent

The authors declare that written informed consent was obtained from all the patients for the publication of this manuscript and accompanying images and attest that the form used to obtain consent from the patients complies with the Journal requirements as outlined in the author guidelines.

## Conflicts of Interest

The authors declare no conflicts of interest.

## Data Availability

The data that support the findings of this study are available on request from the corresponding author. The data are not publicly available due to privacy or ethical restrictions.
